# Influence of Inoculating *Saccharomyces cerevisiae* and *Levilactobacillus brevis* on the Quality of Fermented Large Yellow Croaker (*Larimichthys crocea*): Biogenic Amines, Volatile Components, and Microbial Communities Changes

**DOI:** 10.3390/foods14213690

**Published:** 2025-10-29

**Authors:** Junjie Wu, Na Lin, Jia Yang, Xiujie Zhang, Kaixin Wu, Xinling You, Quanyou Guo

**Affiliations:** 1 East China Sea Fisheries Research Institute, Chinese Academy of Fisheries Sciences, Shanghai 200090, China; wujj@ecsf.ac.cn (J.W.); lina903368043@163.com (N.L.);; 2 College of Ocean Studies, Ningde Normal University, Ningde 352100, China; 3 School of Health Science and Engineering, University of Shanghai for Science and Technology, Shanghai 200093, China; 4 College of Food Science and Technology, Shanghai Ocean University, Shanghai 201306, China; 5 Sandugang Ocean Food Co., Ltd., Ningde 352100, China

**Keywords:** fermented large yellow croaker, *Saccharomyces cerevisiae*, *Levilactobacillus brevis*, biogenic amines, volatile components, microbial composition

## Abstract

As a representative of traditional Chinese fermented fish, fermented large yellow croaker (*Larimichthys crocea*) is characterized by its rich aroma, appealing color, distinctive flavor, and long shelf life. In this study, *Saccharomyces cerevisiae* (*S. cerevisiae*) and *Levilactobacillus brevis* (*L. brevis*) were inoculated to enhance fermentation, and their effects on biogenic amine (BA) formation, volatile flavor compounds (VFCs), and microbial community composition were investigated. Combined inoculation significantly reduced most BAs and nitrite levels, thereby improving the safety of fermented large yellow croaker. The *S. cerevisiae*-inoculated group exhibited higher contents of ester compounds such as 3-methylbutyl acetate, 2-methylpropyl acetate, and ethyl 2-methylbutanoate. *L. brevis* effectively suppressed spoilage and amine-producing bacteria, including *Aeromonas*, *Shewanella*, and *Morganella*. Pearson’s correlation analysis revealed that *Levilactobacillus* was positively correlated with butyl 2-methylbutanoate, (Z)-4-heptenal, pentyl acetate, 2-ethyl-3-methylpyrazine, 3-methylbutyl acetate, 4-methyl-2-pentanone, 3-pentanone, and propanal, while *Saccharomyces* showed positive correlations with propionaldehyde, 1-penten-3-ol, 4-methyl-3-penten-2-one, (E)-2-pentenal, and 2-hexenal. In contrast, BAs were negatively correlated with *Levilactobacillus* and *Saccharomycopsis*, but positively correlated with *Aeromonas*, *Shewanella*, *Photobacterium*, *Vagococcus*, *Vibrio*, *Morganella*, *Lactococcus*, *Apiotrichum*, and *Cutaneotrichosporon*. Overall, these findings demonstrate that *L. brevis* and *S. cerevisiae* improve both the flavor and safety of fermented large yellow croaker. This study offers a theoretical basis for advancing industrial production of fermented fish products.

## 1. Introduction

Blue foods, particularly those derived from marine resources, play a crucial role in the economies, livelihoods, nutritional security, and cultural heritage of many countries [1]. As one of the most important marine fish species in China, the annual production of the *large yellow croaker* (*Larimichthys crocea*) has exceeded 300,000 tons [2], and continues to increase each year [3]. Fermented large yellow croaker, a solid-state fermented product originating from China’s southeastern coastal regions, is well known for its rich aroma, attractive color, distinctive flavor, and long shelf life. Currently, fermented large yellow croaker is primarily produced through traditional, family-based manual methods, resulting in considerable variability in product quality and safety [4]. Moreover, large-scale industrial production often relies on curing rather than true fermentation, leading to a loss of the product’s characteristic flavor and failing to meet consumer expectations.

Flavor is a crucial indicator of fermented food quality. Volatile flavor compounds (VFCs), including aldehydes, ketones, esters, ethers, and organic acids, play a significant role in determining food aroma. The accumulation and transformation of VFCs metabolized by microorganisms are key processes during the late fermentation stage [5]. As an important class of VFCs in fermented fish products, esters have a vital influence on flavor quality [6]. Esters such as propyl butanoate, ethyl acetate, and isobutyl acetate are major contributors to the characteristic fruity and sweet aromas of fermented fish [4,6]. However, undesirable microbial metabolism can lead to the production of harmful compounds, including biogenic amines (BAs) and nitrite. In fermented fish, excessive BAs are not only toxic but also serve as important indicators of deteriorated quality and safety [7]. BAs can react with nitrite to form nitrosamines, which are known carcinogens associated with various types of cancer [8]. Microbial activity is the primary factor influencing the formation of BAs and nitrite in fermented fish products. In particular, bacteria, such as *Hafnia alvei*, *Photobacterium phosphoreum*, and *Morganella morganii* [9], actively generate BAs and nitrite in fermented fish.

To address these challenges, inoculation with starter cultures is a well-established strategy for improving product quality [10]. *Lactobacillus*, *Saccharomyces*, *Staphylococcus*, and *Pediococcus* species, among others, have been widely employed as starter cultures in fermented fish production [7,11]. In our previous work, we isolated lactic acid bacteria (LAB), including *Levilactobacillus brevis* (*L. brevis*) and *Lactiplantibacillus plantarum* (*L. plantarum*), as well as *Saccharomyces cerevisiae* (*S. cerevisiae*), from traditionally fermented *large yellow croaker*. These microorganisms collectively enhance the aroma profile while suppressing the formation of BAs in the fermented product. LAB, often dominant in fermentation systems, plays a crucial role in shaping flavor profiles through the production of VFCs and free amino acids, while simultaneously inhibiting spoilage microorganisms [5]. Complementing the activity of LAB, yeasts such as *Wickerhamomyces anomalus* and *S. cerevisiae* enrich the diversity of VFCs by metabolizing fish lipids into unsaturated fatty acid precursors of key aroma compounds [12,13]. This co-culture system has been shown to significantly reduce the formation of *tyramine* in fermented fish [12], thereby enhancing product safety. However, the critical safety implications of such dynamic co-fermentation environments remain largely unassessed in fermented *large yellow croaker*. Moreover, few studies have comprehensively investigated the microbial composition, flavor profiles, and safety parameters during its fermentation process.

In this study, fermented *large yellow croaker* was used as the substrate to evaluate the effects of individual and mixed inoculations of *L. brevis* and *S. cerevisiae* on the fermentation process. Dynamic changes in physicochemical properties, safety parameters, VFCs, and microbial community structure were monitored throughout fermentation. The primary objective was to investigate the influence of bacterial and yeast inoculation on the safety and quality of fermented *large yellow croaker*, as well as to explore the relationships between microbial communities and flavor formation. This study is expected to improve the fermentation safety and quality of fermented large yellow croaker, promote its standardized production, and facilitate the application of commercial starters for fermented fish.

## 2. Materials and Methods

### 2.1. Materials and Chemicals

Large yellow croakers were obtained from Ningde Cai Shi Aquatic Co., Ltd. (Ningde, China). The fish were scaled, eviscerated, split along the back, washed with water, vacuum-packed, and transported to the laboratory in an icebox within 24 h. *Hong Qu* lees were prepared by filtering *Hongqu* glutinous rice wine produced in our laboratory [14] and stored at 4 °C. *L. brevis* (strain LB) and *S. cerevisiae* (strain SC) were isolated from traditionally fermented fish. All analytical-grade chemicals were purchased from Sinopharm Chemical Reagent Co., Ltd. (Shanghai, China).

### 2.2. Preparation of Strains

*L. brevis* was sub-cultured twice in MRS broth, while *S. cerevisiae* was sub-cultured twice in PDA broth. The cultures were centrifuged at 10,000 r/min for 10 min at 4 °C, washed twice with sterile saline solution (0.85% NaCl), and resuspended to a final concentration of 10^7^ CFU/mL. The strain suspensions were stored at 4 °C for immediate use.

### 2.3. Preparation of Samples

Large yellow croakers (400 ± 25 g, *n* = 60) were immersed in a brine solution consisting of 9:100 (salt to fish, *g*/*g*), 9:1000 (sugar to fish, *g*/*g*), 3:500 (Ajinomoto to fish, *g*/*g*), and 3:100 (salt to water, *w*/*v*) at 4 °C for 12 h, and then air-dried. The fish were subsequently placed on shelves and dried at 20 ± 2 °C and 45% relative humidity (RH) in a constant-temperature heat pump dryer (CFZD-10/S, Shandong Paifeike Industrial Equipment Co., Ltd., Weifang, China) until the moisture content reached 45 ± 2%. Based on our previous study, microwave treatment combined with slightly acidic electrolyzed water was applied to reduce the bacterial load [15]. The fish were then mixed with 10% *Hong Qu* lees (*g*/*g*) and 4% strain cultures (*w*/*v*), vacuum-packed, and incubated in a constant-temperature hot-air oven (PH-070(A), Bluepard Instruments, Shanghai, China) at 20 °C for 8 days. Four treatment groups were prepared: CK (natural fermentation), LB (*L. brevis* inoculated), SC (*S. cerevisiae* inoculated), and LS (*L. brevis* + *S. cerevisiae* co-culture, 1:1 ratio). All samples (*n* = 3 per batch, skinless back muscle) were collected on days 0, 2, 4, 6, and 8 for subsequent analyses and stored at −80 °C until use.

### 2.4. Determination of Microbial Counts

Samples (10 g) were aseptically mixed with 90 mL of sterile saline solution (0.85% NaCl) and homogenized for 2 min. Appropriate decimal dilutions were then prepared using the same saline solution. Total aerobic bacterial counts were determined on Plate Count Agar (Qingdao Hope Bio-Technology Co., Ltd., Qingdao, China) after incubation at 28 °C for 72 h. Yeast counts were determined on YPD agar medium under the same incubation conditions. LAB counts were determined on MRS agar medium following anaerobic incubation at 37 °C for 72 h.

### 2.5. Determination of Total Acidity (TA), Total Volatile Base Nitrogen (TVB-N) and Thiobarbituric Acid Reactive Substances (TBARS)

The determination of TA was performed according to the method described by Qian et al. [16]. TA was measured using a digital burette (Titrette, Brand, Germany) and expressed as lactic acid content. The TVB-N content was determined following the method of Zhang et al. [17] using an automatic Kjeldahl apparatus (KDN-103F, Shanghai Xianjian Instrument Co., Ltd., Shanghai, China). TBARS were analyzed according to the method of Li et al. [18] with slight modifications. A 5 g sample from each treatment was homogenized with 50 mL of 7.5% TCA solution for 30 min. After cooling, the homogenate was filtered through double-layer filter paper, and the filtrate was collected. Subsequently, 1 mL of a 0.02 M TBA solution was added to an equal volume of filtrate and mixed. The mixture was in a water bath maintained at 90 °C for 30 min and then cooled in ice water. The absorbance was measured at 532 nm, and a standard curve was plotted using 1,1,3,3-tetramethoxypropane.The results were expressed as mg of malondialdehyde (MDA) per kg of sample.

### 2.6. Determination of BAs and Nitrite

The determination of BAs was conducted according to the method described by Li et al. [19], with slight modifications. Briefly, 10.0 g of the sample was homogenized with 20 mL of 5% TCA for 30 min. The homogenate was centrifuged at 5000 rpm for 10 min, and the supernatant was collected. The residue was re-extracted with 5% TCA, and the combined supernatants were adjusted to a final volume of 50 mL. Derivatization of the extracts was performed following the method of Yang et al. [20]. The derivatized samples were filtered through a 0.22 μm nylon membrane filter and transferred into 2 mL vials for analysis. BAs were quantified using a high-performance liquid chromatography (HPLC) system (1260, Agilent Technologies, Santa Clara, CA, USA) equipped with a SB-C18 column (4.6 mm × 250 mm, 5 μm) and an ultraviolet (UV) detector (G1314F, Agilent Technologies, Santa Clara, CA, USA). The concentrations of individual BAs were calculated using calibration curves generated from standard solutions of known concentrations. Nitrite content was determined according to the method described by Jiao et al. [21].

### 2.7. Determination of VFCs

VFCs in fermented large yellow croaker were analyzed using gas chromatography-ion mobility spectrometry (GC-IMS) (FlavourSpec^®^, Gesellschaft für Analytische Sensorsysteme mbH, Dortmund, Germany) [6]. Approximately 0.5 g of the sample was placed into a 20 mL headspace vial for automated sampling. The incubation temperature was set to 40 °C for 15 min. Headspace injection was performed with oscillation heating at 500 rpm, an injection temperature of 65 °C, and an injection volume of 500 μL. The gaseous sample was separated using an MXT-WAX capillary column (30 m × 0.53 mm, 1 μm film thickness). The column temperature was maintained at 60 °C, and high-purity nitrogen (≥99.999%) was used as the carrier gas. The carrier gas flow program was as follows: 2.0 mL/min for 2 min, increased to 10.0 mL/min over 8 min, then to 100.0 mL/min over 10 min, and finally held at 100.0 mL/min for 10 min. The total analysis time was 30 min.

### 2.8. DNA Extraction and PCR Amplification

Skinless back muscle samples were collected for microbial community analysis, with three biological replicates from each treatment group. DNA was extracted using the Mag-Bind^®^ Soil DNA Kit (Omega Bio-Tek, Norcross, GA, USA). The concentration and purity of the extracted DNA were determined using a NanoDrop 2000 spectrophotometer (Thermo Fisher Scientific, Waltham, MA, USA), and DNA integrity was evaluated by 1% agarose gel electrophoresis. The bacterial 16S rRNA gene was amplified using primers 338F (5′-ACCTACGGGAGGCAGCAG-3′) and 806R (5′-GGACTACHVGGGTWTCTAAT-3′), while the fungal ITS rRNA gene was amplified with primers ITS1F (5′-CTTGGTCATTTAGAGGAAGTAA-3′) and ITS2R (5′-GCTGCGTTCTTCATCGATGC-3′). PCR products were purified using an AxyPrep DNA Gel Extraction Kit (Axygen, Union City, CA, USA), and the purified DNA was quantified with a QuantiFluor™-ST system (Promega, Madison, WI, USA).

Purified amplicons were pooled in equimolar amounts and subjected to paired-end sequencing on an Illumina NextSeq 2000 platform (Illumina, San Diego, CA, USA) following the standard protocols of Majorbio Bio-Pharm Technology Co., Ltd. (Shanghai, China).

To minimize the influence of sequencing depth on alpha and beta diversity analyses, sequence data were rarefied to 24,520 reads for the 16S rRNA gene and 29,027 reads for the ITS rRNA gene, which yielded an average Good’s coverage of 99.99%.

### 2.9. Data Analysis

All statistical analyses were performed using the mean values of three biological replicates. The data were analyzed by one-way analysis of variance (ANOVA) using SPSS 27.0 (IBM, Armonk, NY, USA), and significant differences among means were determined by Tukey’s multiple comparison test (*p* < 0.05). Orthogonal partial least squares discriminant analysis (OPLS-DA) was conducted using SIMCA 14.1 (Umetrics, Umeå, Sweden). Fingerprints of VFCs were constructed using the Reporter plug-in in LAV (Laboratory Analytical Viewer), where “M” and “D” denote monomer and dimer signals, respectively. Bar charts and correlation plots were generated using OriginPro 2021 (OriginLab, Northampton, MA, USA). Non-metric multidimensional scaling (NMDS) and Upset plot analyses were performed using R software (version 4.4.1).

## 3. Results and Discussion

### 3.1. Growth of Microbial Counts

**Figure 1:**
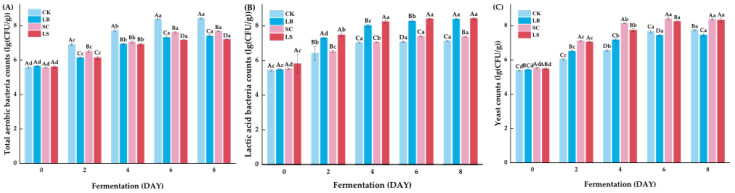
Microbial dynamics during the fermentation of large yellow croaker. (**A**) Total aerobic bacteria counts; (**B**) lactic acid bacteria (LAB) counts; (**C**) yeast counts. Treatment groups: CK: natural fermentation, LB: *L. brevis* inoculated, SC: *S. cerevisiae* inoculated, LS: *L. brevis* and *S. cerevisiae* co-culture. ^a–d^ Values in the same group with different letters were significantly different (*p* < 0.05), while ^A–D^ Values on the same day with different letters were significantly different (*p* < 0.05).

As shown in Figure 1A–C, the microbiological dynamics during the fermentation of large yellow croaker were evaluated with and without the addition of starter cultures. Total aerobic bacterial counts exhibited an initial increasing trend in all treatment groups. The CK group (natural fermentation) showed a faster increase, reaching 8.38 log CFU/g on day 6. However, no significant difference (*p* > 0.05) was observed between days 6 and 8 within the same group. A similar pattern was observed for LAB, where the counts exceeded 8 log CFU/g in both LB and LS groups by day 4. Notably, the LS group exhibited significantly higher LAB counts (*p* < 0.05) than the LB group on days 6 and 8. These results are consistent with the findings of Zhang et al. [22], who reported that the inoculation of mixed strains (*L. plantarum* and *S. cerevisiae*) significantly increased LAB counts during *Suanyu* fermentation. The yeast population in the SC and LS groups increased from an initial 5.49–5.52 log CFU/g to 8.24–8.40 log CFU/g by day 6, whereas those in the other groups reached only about 7 log CFU/g. In contrast to the LAB trend, the yeast population stabilized after day 6, suggesting that nutrient depletion and environmental stress (e.g., low pH, oxygen limitation) may have inhibited further growth.

### 3.2. Physiochemical Parameters of Fermented Large Yellow Croaker

**Figure 2:**
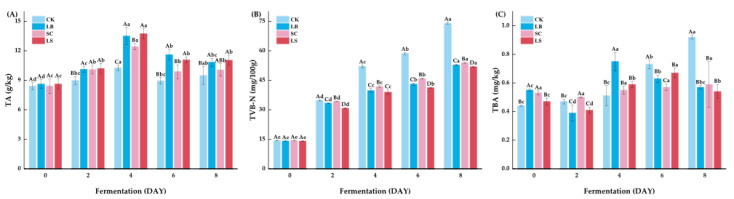
Physicochemical indexes during fermentation of fermented large yellow croaker. (**A**) TA, (**B**) TVB-N, (**C**) TBA. Treatment groups: CK: natural fermentation, LB: *L. brevis* inoculated, SC: *S. cerevisiae* inoculated, LS: *L. brevis* and *S. cerevisiae* co-culture. ^a–e^ Values in the same group with different letters were significantly different (*p* < 0.05), while ^A–D^ Values on the same day with different letters were significantly different (*p* < 0.05).

As shown in Figure 2A, TA values of all samples increased sharply to 10.26–13.74 after four days of fermentation, followed by a slight stabilization during the subsequent two days. No significant difference (*p* > 0.05) was observed between the LB and LS groups, suggesting that yeast addition did not significantly affect the acidification dynamics or compromise the antimicrobial efficacy against spoilage and pathogenic bacteria [12].

TVB-N serves as an important indicator of fish freshness and the degree of spoilage. As shown in Figure 2B, no significant difference (*p* > 0.05) was observed among the three groups at the beginning of fermentation. However, all groups exhibited a significant increase (*p* < 0.05) after two days. By day 8, the inoculated groups showed approximately 30% lower TVB-N levels than the CK group, indicating a stronger inhibitory effect on spoilage bacteria [23].

TBA are commonly used to evaluate lipid oxidation in fermented products [24]. As depicted in Figure 2C, TBARS values in the CK group increased from 0.44 mg/kg to 0.92 mg/kg during the 8-day fermentation period. In contrast, the starter-culture-inoculated groups reached maximum values of approximately 0.60 mg/kg, indicating that lipid oxidation levels remained acceptable. According to previous studies, fish muscle typically develops off-odors when TBARS values exceed 1 mg/kg [25]. Our findings were consistent with TBARS levels in wine lees golden pomfret [26].

### 3.3. Safety Parameters of Fermented Large Yellow Croaker

BAs are among the most important food safety indicators in fermented foods [27]. A total of seven BAs, including tryptamine, phenylethylamine, putrescine, cadaverine, histamine, tyramine and spermidine (Table 1), were detected in fermented large yellow croaker. Among these, histamine is recognized as the most toxic BA, commonly found in fermented fish products, followed by tyramine [28]. In the CK group, histamine concentrations increased to 53.96 and 66.69 mg/kg on days 6 and 8, respectively, indicating potential safety risks. According to U.S. regulations, the permissible limit for histamine in fish products is 50 mg/kg [29]. Notably, the LB group exhibited an 86% reduction in histamine content compared with the CK group at day 8, demonstrating that inoculation with *L. brevis* effectively inhibited histamine-producing bacteria. These results are inconsistent with observations in *Suanyu*, where histamine was undetectable during fermentation [30]. This discrepancy presumably stems from the high histidine content in marine fish muscle, which serves as a precursor for histamine formation [31]. Cadaverine, putrescine, and tryptamine were the most abundant BAs detected in fermented large yellow croaker, consistent with previous findings in other fermented fish products. Compared with the CK group, tryptamine levels in the inoculated fermentation groups were significantly lower (*p* < 0.05) on day 4. Cadaverine and putrescine, typically associated with *Enterobacteriaceae* contamination, contribute to unpleasant off-odors when combined with sulfides, masking desirable aldehyde-derived aromas from lipid oxidation. In this study, inoculation significantly reduced cadaverine concentrations, particularly in the SC group, which showed a 47% reduction relative to the CK group on day 8. In contrast, yeast or LAB inoculation had a limited impact on putrescine levels. Phenylethylamine and spermidine remained at relatively low concentrations (<10 mg/kg). This result differs from a previous study reporting that inoculation with *L. plantarum* reduced putrescine accumulation in low-salt fermented sausages [32], likely due to differences in microbial strains and fermentation conditions. Nitrite can react with amino acids or BAs to form carcinogenic nitrosamines, posing potential health risks [8]. During the first six days of fermentation, nitrite levels in all groups remained low (<1 mg/kg). By day 8, significantly lower nitrite contents (*p* < 0.05) were observed in the inoculated groups compared with CK, indicating that starter cultures effectively reduced nitrite accumulation during the late fermentation stage. Overall, mixed starter culture inoculation effectively inhibited the formation of most BAs and nitrite, thereby improving the safety of fermented large yellow croaker.

### 3.4. VFC Profiles of Fermented Large Yellow Croaker

**Figure 3:**
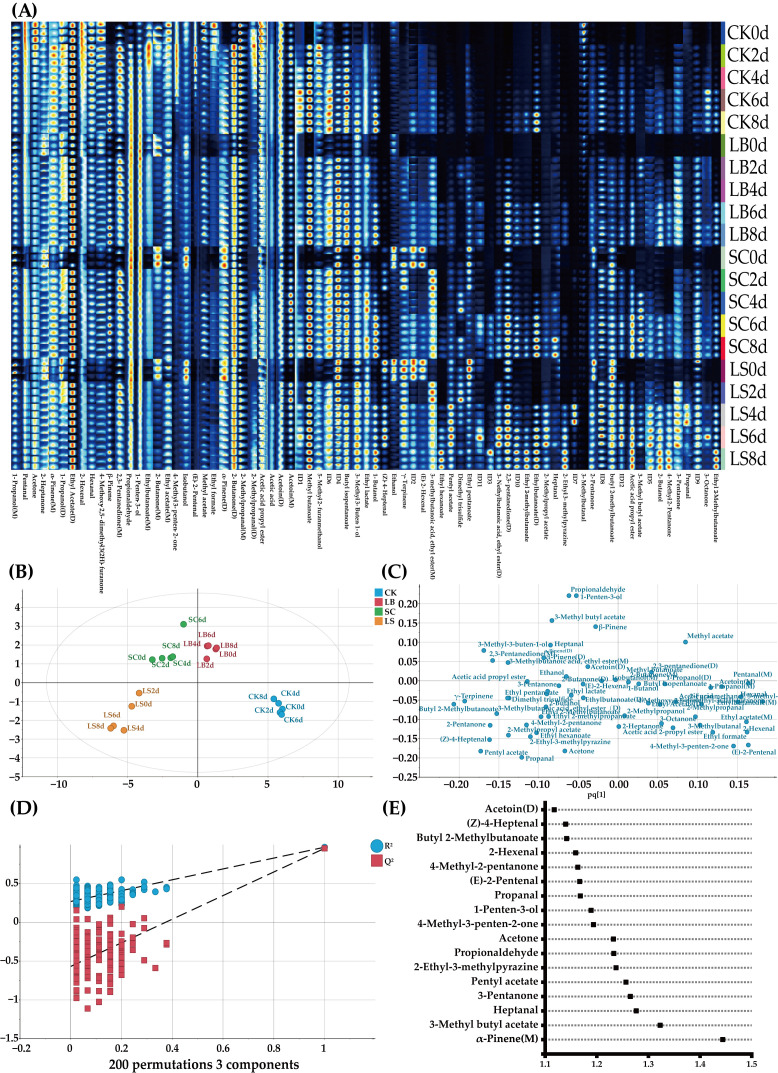
VFCs changes in fermented large yellow croaker with starter cultures during fermentation. (**A**): Fingerprint of volatile components; (**B**): scores plots of OPLS-DA,; (**C**): loading plot of OPLS-DA; (**D**): cross-validation plot by 200 permutation tests; (**E**): key differential compounds with VIP > 1.1. CK: natural fermentation, LB: *L. brevis* inoculated, SC: *S. cerevisiae* inoculated, LS: *L. brevis* and *S. cerevisiae* co-culture, 0d: start of fermentation, 4d: after 4 d of fermentation.

The VFC fingerprints effectively distinguished closely related substances (Figure 3A). Each point represents a volatile organic compound, with the color intensity and area corresponding to its concentration—white indicates low abundance, red indicates higher abundance, and darker shades denote the highest concentrations. Based on comparison and qualitative analysis using the HS-GC-IMS database, a total of 63 VFCs were identified, including their monomeric (M) and dimeric (D) forms, along with 12 unidentified compounds. Several pungent or fishy odor compounds, such as 1-propanol, hexanal, 2-hexenal, and 3-octanone, increased markedly in the CK group during fermentation, indicating a deterioration in odor quality. These compounds are typically generated through amino acid degradation and lipid oxidation processes [22]. Acetic acid, primarily produced by lactic acid bacteria, also increased throughout fermentation in all groups and contributed positively to flavor development, consistent with the observed changes in TA. Esters are the principal contributors to the floral and fruity aroma characteristics of fermented fish products [4]. Compared with the CK group, the SC and LS groups exhibited significantly higher concentrations of esters such as 3-methylbutyl acetate (apple, banana, pear), 2-methylpropyl acetate (apple, banana, herb), ethyl butanoate (apple, butter, cheese, pineapple, strawberry), ethyl 2-methylbutanoate (green apple, kiwifruit, strawberry), and propyl acetate (celery, floral, pear). These results align with Gao et al. [11], who demonstrated that *S. cerevisiae* inoculation significantly enhances ester production in *Suanyu*. Similarly, An et al. reported LAB inoculation in *Zhayu* fermentation accelerates proteolytic activity, with significant increases in alcohol and ester concentrations contributing to enhanced fragrance profiles [33]. Collectively, these findings establish co-culture fermentation as a viable strategy for optimizing flavor quality in fermented fish products.

Effective differentiation of odor profiles in fermented large yellow croaker during fermentation was achieved using OPLS-DA. As shown in Figure 3B, all four sample groups were distinctly clustered, indicating clear separation among fermentation treatments. The fitting indices for the independent (R^2^_X_) and dependent (R^2^_Y_) variables were 0.922 and 0.920, respectively. The predictive capability of the model (Q^2^) was 0.826, demonstrating excellent model reliability and predictive performance [34]. This robust model provides a reliable tool for discriminant analysis across different fermentation matrices. The key discriminant volatile compounds contributing to the variation in aromatic profiles among fermentation batches were identified through interpretation of the OPLS-DA loading plot (Figure 3C). After 200 permutation tests (Figure 3D), the intersection of the Q^2^ regression line with the vertical axis was below zero, confirming that the model was not overfitted and that the validation was effective. The variable importance in projection (VIP) score was used to assess the relative contribution of each compound in the OPLS-DA model, with VIP > 1 generally considered significant [35]. In fermented large yellow croaker, 17 volatile compounds exhibited VIP values greater than 1.1 (Figure 3E), among which α-pinene (M) and 3-methylbutyl acetate showed the highest scores.

### 3.5. Alpha and Beta Diversity of the Microbial Community in Fermented Large Yellow Croaker

To explore the effects of inoculated fermentation on bacterial and fungal communities, 16S and ITS rRNA gene sequencing was conducted on fermented large yellow croaker samples collected on days 0 and 4.

**Figure 4:**
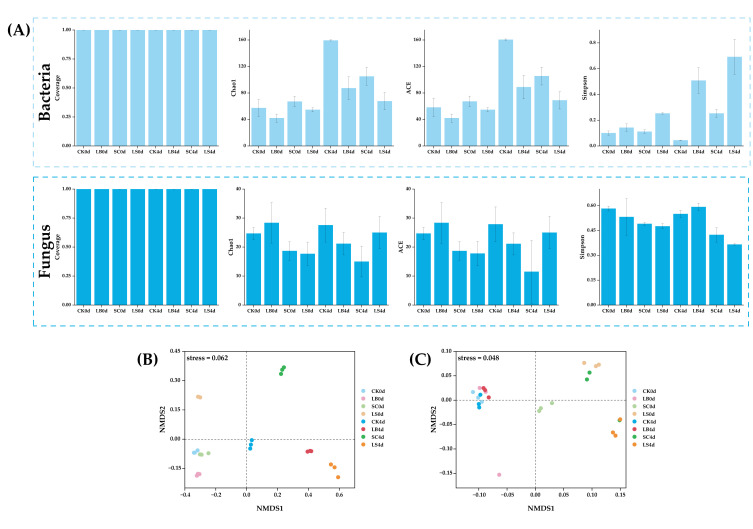
(**A**): The microbial α-diversity. The α-diversity indices included Chao1, ACE, and Simpson indices. The upper row corresponds to bacteria, while the lower row corresponds to fungi. The β-diversity, (**B**) bacteria, (**C**) fungi. CK: natural fermentation, LB: *L. brevis* inoculated, SC: *S. cerevisiae* inoculated, LS: *L. brevis* and *S. cerevisiae* co-culture, 0d: start of fermentation, 4d: after 4 d of fermentation.

As shown in Figure 4A, alpha diversity indices were used to assess microbial community richness and diversity. The Chao1 and ACE indices reflect microbial abundance, with higher values indicating richer communities [6], whereas higher Simpson index values correspond to greater diversity [36]. At day 0, bacterial richness and diversity were at their lowest levels. By day 4, the LB4d, SC4d, and LS4d groups exhibited lower Chao1 and ACE values than the CK4d group, while their Simpson indices were higher. These results suggest that inoculated fermentation reduced overall species richness but enhanced evenness, particularly in the LS4d group. For the fungal community, richness increased in the CK and LS groups, whereas a decreasing trend was observed in the LB and SC groups. The Simpson index values of the SC and LS groups decreased, indicating higher fungal diversity after inoculation.

Beta diversity was visualized using NMDS to illustrate the differences in microbial community composition during fermentation. The distribution of samples along the NMDS axes revealed overall patterns of community change [9]. NMDS analysis based on bacterial OTUs (Figure 4B) showed that all groups were closely clustered at day 0 but became more dispersed by day 4, indicating increasing differentiation among microbial communities. As illustrated in Figure 4C, fungal OTUs in the CK and LB groups exhibited minimal temporal variation, remaining closely clustered throughout fermentation. In contrast, the SC and LS groups displayed progressive temporal divergence, suggesting that yeast inoculation substantially altered the fungal community structure during fermentation.

### 3.6. Microbial Community Composition in Fermented Large Yellow Croaker

**Figure 5:**
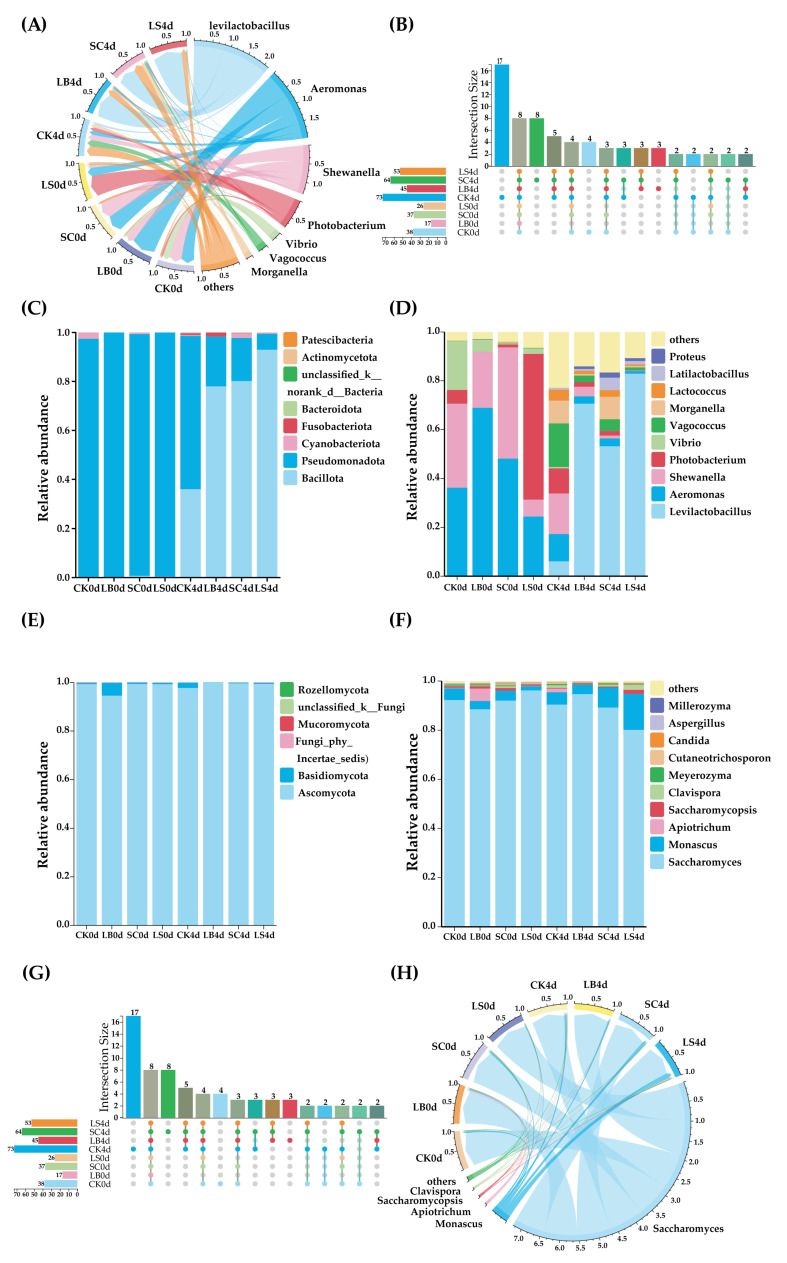
The chord diagram, (**A**) bacteria, (**H**) fungi. Relative abundance of bacterial (**C**) and fungal (**E**) composition in fermented large yellow croaker at the phylum level. Relative abundance of bacterial (**D**) and fungal (**F**) composition in fermented large yellow croaker at the gene level. The UpSet plot, (**B**) bacteria, (**G**) fungi. CK: natural fermentation, LB: *L. brevis* inoculated, SC: *S. cerevisiae* inoculated, LS: *L. brevis* and *S. cerevisiae* co-culture, 0d: start of fermentation, 4d: after 4 d of fermentation.

Analysis of OTU data revealed distinct microbial diversity patterns across fermentation time points. UpSet plots were used to visualize the distribution of unique and shared OTUs among the inoculated treatment groups (Figure 5B,G). Bacterial communities differed markedly among treatments, with 73, 45, 64, and 53 OTUs detected in the CK4d, LB4d, SC4d, and LS4d groups, respectively. Five OTUs were common to all four groups, suggesting that these shared taxa may play essential roles in the fermentation process of the large yellow croaker. For fungi, eight unique core genera were identified in the CK0d group. The LB4d and SC4d groups each contained four unique core genera, whereas the CK4d and LS4d groups contained two. Two core fungal genera were shared among all samples.

The bacterial phyla identified are shown in Figure 5C. *Pseudomonadota* (formerly *Proteobacteria*) was the dominant phylum in fish samples at day 0, accounting for 97% of the total bacterial community. This finding agrees with previous reports showing that the relative abundance of *Pseudomonadota* increased after microwave pretreatment in fermented large yellow croaker sauce before fermentation [6]. After four days of fermentation, *Bacillota* (formerly *Firmicutes*) became the predominant phylum in the inoculated groups. Similar trends have been observed in inoculated fermentations of *Suanyu* [37] and *Zhayu* [4]. Figure 5D presents the top 10 bacterial genera by relative abundance. The key bacterial taxa identified included *Levilactobacillus*, *Aeromonas*, *Shewanella*, *Photobacterium*, *Vibrio*, *Vagococcus*, and *Morganella* (Figure 5A). At day 0, the dominant genera were *Aeromonas* (24.34–68.90%), *Shewanella* (6.96–45.61%), *Photobacterium* (0.02–59.72%), and *Vibrio* (2.28–5.10%). These bacteria likely originated from pre-processing steps such as salting and drying, as reported by Shen et al. [38]. They are known spoilage organisms in fish products [39], responsible for producing off-flavors and generating Bas [5]. After fermentation, *Levilactobacillus* became the dominant bacterial genus. Its relative abundance reached 82.92% in the LS4d group, compared with only 6.11% in the CK4d group. Lactic acid bacteria (LAB) inhibit the growth of spoilage microorganisms through acid production, consistent with the increased TA observed. Interestingly, *Levilactobacillus* also dominated in the SC4d group (53.16%), while the CK4d group remained dominated by *Aeromonas* (11.15%), *Shewanella* (16.53%), and *Vagococcus* (17.84%). This indicates that *S. cerevisiae* inoculation altered the relative abundance of dominant bacterial genera in fermented large yellow croaker. Previous studies have shown that *S. cerevisiae* can inhibit pathogenic and spoilage bacteria by producing ethanol, short-chain fatty acids, and antimicrobial peptides [36]. *Vagococcus*, a microorganism associated with BA formation in fermented tilapia surimi [27], showed a sharp increase in abundance by day 4. *Morganella*, a known histamine-producing bacterium [9], reached approximately 9% relative abundance in the CK4d and SC4d groups but remained below 1% in the LB4d and LS4d groups. This suggests that a high abundance of *Levilactobacillus* may effectively suppress *Morganella* growth.

Figure 5E,F depict the relative abundances of all detected fungal phyla and the top 10 fungal genera, respectively. *Ascomycota* dominated the fungal community throughout fermentation, accounting for more than 94% of the total relative abundance at all time points. A similarly high abundance of *Ascomycota* (97.05%) was reported in *Zaoyu* fermented with *Chuzhai* starter by Nie et al. [40]. In contrast, *Basidiomycota* exhibited an increasing trend in the CK group, reaching approximately 2% at day 4, while in the inoculated groups, its abundance decreased to below 0.5%. The predominant fungal genera identified were *Saccharomyces* and *Monascus* (Figure 5H). *Saccharomyces* was the most abundant fungus, accounting for more than 80% of the fungal community. This genus contributes to flavor enhancement by decomposing fatty acids and improving the nutritional quality of fermented fish through optimization of phospholipid molecular composition [5]. *Monascus*, a dominant fungal genus in *Hongqu*-fermented products such as surimi [41], rice wine [14], and fish bone [42], was also detected. Known for producing various bioactive metabolites beneficial to human health, *Monascus* initially represented 1.73–4.73% of the community and increased to 3.87–14.60% by day 4, with the highest abundance observed in the LS4d group.

### 3.7. Correlation of Microorganisms and Metabolites

**Figure 6:**
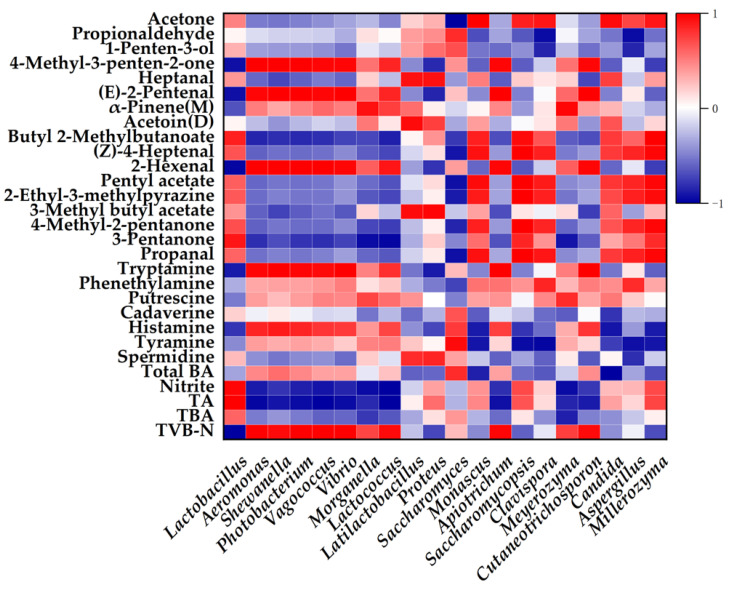
Correlations between microorganisms and detection indexes. Positive (0 < r < 1) and negative (−1 < r < 0) correlations are indicated in red and blue, respectively.

To further investigate the relationships between dominant microbial communities and key physicochemical indicators, Pearson’s correlation analysis was performed using the relative abundances of the top 10 bacterial and fungal genera, along with volatile compounds (VIP > 1.1) and major non-volatile indicators (Figure 6).

The analysis revealed that *Levilactobacillus*, *Monascus*, *Saccharomycopsis*, *Clavispora*, *Candida*, *Aspergillus*, and *Millerozyma* were positively correlated with multiple VFCs, including butyl 2-methylbutanoate, (Z)-4-heptenal, pentyl acetate, 2-ethyl-3-methylpyrazine, 3-methylbutyl acetate, 4-methyl-2-pentanone, 3-pentanone, and propanal. These VFCs were abundant in the SC and LS groups, consistent with previous findings in fermented fish products [5]. Ester biosynthesis relies primarily on the synergistic interplay of esterification and alcoholysis [20]. *Levilactobacillus* contributes to ester formation through its acid-producing ability, while fungi are enabled through their metabolic capacity to generate alcohols [40]. Aldehydes and ketones arise primarily from the oxidation of unsaturated fatty acids and the catabolic breakdown of amino acids [43]. *Levilactobacillus* has been reported to exhibit significant capabilities in amino acid metabolism and lipid metabolism [44]. Notably, 2-ethyl-3-methylpyrazine has been identified as a pivotal flavor substance in *Zhayu* products [4], and its formation is commonly attributed to Strecker degradation [45]. *Aspergillus* and *Bacillus* have the ability to produce pyrazine during the fermentation of fermented soybean product [45]. Conversely, these compounds were negatively correlated with the relative abundances of *Aeromonas*, *Shewanella*, *Photobacterium*, *Vagococcus*, *Vibrio*, *Morganella*, and *Lactococcus*. In addition, *Saccharomyces* exhibited positive correlations with propionaldehyde, 1-penten-3-ol, 4-methyl-3-penten-2-one, (E)-2-pentenal, and 2-hexenal. Notably, 1-penten-3-ol has been identified as a key contributor to the characteristic aroma of fermented large yellow croaker sauce [15] and *Zaoyu* (mackerel) [46].

Among non-volatile safety indicators, tryptamine, putrescine, histamine, and tyramine were negatively correlated with *Levilactobacillus* and *Saccharomycopsis*, but positively correlated with *Aeromonas*, *Shewanella*, *Photobacterium*, *Vagococcus*, *Vibrio*, *Morganella*, *Lactococcus*, *Apiotrichum*, and *Cutaneotrichosporon*. Although several LAB, including *Levilactobacillus*, *Lactococcus*, and *Streptococcus*, have been reported to contribute to BA formation in fermented fish products [31], Lee et al. [47] demonstrated that *L. brevis* PK08 can degrade BAs through the expression of a multicopper oxidase gene. Furthermore, *Aeromonas*, *Shewanella*, *Photobacterium*, *Vagococcus*, *Vibrio*, *Morganella*, *Lactococcus*, *Apiotrichum*, *Meyerozyma*, and *Cutaneotrichosporon* showed negative correlations with TA and positive correlations with TVB-N, suggesting that their proliferation may be associated with quality deterioration during fermentation.

However, although heat maps identify potential microbial-metabolite associations, these correlations do not imply direct causation and may reflect indirect ecological or temporal linkages. Future studies will integrate metagenomics and metabolomics to conduct functional validation, perform pathway analysis, build biological network models, and implement experimental verification to establish causal “organism → compound” links.

## 4. Conclusions

This study demonstrated that inoculation with *L. brevis* and *S. cerevisiae* promoted the growth of LAB and accelerated the fermentation process of the large yellow croaker. The LS group exhibited approximately 34% higher TA and 25% lower TVB-N compared with the CK group. Combined inoculation significantly reduced the formation of most BAs and nitrite, thereby enhancing product safety. The *S. cerevisiae*-inoculated samples contained higher levels of ester compounds, including 3-methylbutyl acetate, 2-methylpropyl acetate, and ethyl 2-methylbutanoate, contributing to improved aroma quality. LAB, as the dominant bacterial group, effectively inhibited spoilage and amine-producing bacteria such as *Aeromonas*, *Shewanella*, and *Morganella*, thereby improving overall product quality. Correlation analysis revealed that *Levilactobacillus* was positively associated with butyl 2-methylbutanoate, (Z)-4-heptenal, pentyl acetate, 2-ethyl-3-methylpyrazine, 3-methylbutyl acetate, 4-methyl-2-pentanone, 3-pentanone, and propanal, while *Saccharomyces* correlated positively with propionaldehyde, 1-penten-3-ol, 4-methyl-3-penten-2-one, (E)-2-pentenal, and 2-hexenal. In contrast, BAs showed negative correlations with *Levilactobacillus* and *Saccharomycopsis*, but positive correlations with *Aeromonas*, *Shewanella*, *Photobacterium*, *Vagococcus*, *Vibrio*, *Morganella*, *Lactococcus*, *Apiotrichum*, and *Cutaneotrichosporon*. Overall, these findings confirm that *L. brevis* and *S. cerevisiae* improve both the flavor and safety of fermented large yellow croaker. Further studies should focus on starter culture standardization and pilot-scale fermentation applications and elucidating the metabolic pathways through which *L. brevis* and *S. cerevisiae* regulate volatile compound and BAs formation.

## Figures and Tables

**Table 1:** The accumulation of BAs (mg/kg) and nitrite (mg/kg) of fermented large yellow croaker during fermentation.

Index	Days	CK	LB	SC	LS
Tryptamine	0	2.75 ± 0.52 ^Be^	3.41 ± 0.01 ^Ae^	3.45 ± 0.03 ^Ad^	2.13 ± 0.15 ^Cc^
	2	5.64 ± 0.02 ^Ad^	3.55 ± 0.17 ^Bd^	1.49 ± 0.10 ^De^	2.66 ± 0.01 ^Cc^
	4	28.99 ± 0.02 ^Ab^	17.68 ± 0.06 ^Bc^	15.32 ± 0.29 ^Dc^	16.21 ± 0.23 ^Cb^
	6	25.56 ± 0.06 ^Bc^	30.31 ± 1.12 ^Aa^	16.48 ± 0.50 ^Db^	21.04 ± 2.86 ^Ca^
	8	45.60 ± 0.24 ^Aa^	20.08 ± 0.19 ^Db^	37.57 ± 0.24 ^Ba^	21.79 ± 0.42 ^Ca^
Phenethylamine	0	1.64 ± 0.08 ^Bd^	1.73 ± 0.10 ^Ad^	1.76 ± 0.02 ^Ad^	1.57 ± 0.03 ^Bd^
	2	1.18 ± 0.05 ^Be^	1.29 ± 0.01 ^Ad^	1.18 ± 0.05 ^Be^	1.18 ± 0.26 ^Be^
	4	2.87 ± 0.34 ^Bc^	2.25 ± 0.16 ^Dc^	2.43 ± 0.02 ^Cc^	2.90 ± 0.03 ^Ac^
	6	7.47 ± 0.22 ^Ab^	4.85 ± 0.02 ^Cb^	5.64 ± 0.54 ^Bb^	5.50 ± 0.28 ^Bb^
	8	9.30 ± 0.06 ^Aa^	8.06 ± 0.62 ^Ba^	7.19 ± 0.03 ^Ca^	6.66 ± 0.10 ^Ca^
Putrescine	0	9.95 ± 1.00 ^Ae^	8.91 ± 0.89 ^Be^	10.69 ± 0.02 ^Ae^	8.53 ± 0.29 ^Be^
	2	13.75 ± 0.01 ^Cd^	19.82 ± 0.56 ^Ad^	14.19 ± 0.32 ^Cd^	16.27 ± 0.01 ^Bd^
	4	41.95 ± 0.04 ^Ac^	23.67 ± 0.04 ^Cc^	41.56 ± 0.63 ^Ac^	37.81 ± 0.44 ^Bc^
	6	58.01 ± 1.94 ^Ab^	49.15 ± 0.05 ^Bb^	49.68 ± 1.26 ^Bb^	52.47 ± 3.17 ^Bb^
	8	87.19 ± 0.63 ^Aa^	75.48 ± 0.52 ^Ba^	87.45 ± 0.42 ^Aa^	71.69 ± 1.23 ^Ca^
Cadaverine	0	7.95 ± 0.71 ^Be^	6.40 ± 0.01 ^Ce^	10.86 ± 0.02 ^Ad^	6.53 ± 0.19 ^Cd^
	2	49.89 ± 0.03 ^Dd^	70.93 ± 2.01 ^Cd^	79.92 ± 1.81 ^Bc^	92.93 ± 0.17 ^Ac^
	4	101.61 ± 0.04 ^Bc^	151.75 ± 0.15 ^Aa^	82.81 ± 1.24 ^Dc^	91.12 ± 1.13 ^Cc^
	6	149.07 ± 0.33 ^Ab^	139.86 ± 4.64 ^Bb^	131.79 ± 3.54 ^Bb^	104.63 ± 6.67 ^Cb^
	8	173.14 ± 1.58 ^Aa^	114.68 ± 1.12 ^Cc^	92.47 ± 0.52 ^Da^	118.39 ± 1.99 ^Ba^
Histamine	0	14.69 ± 1.48 ^Be^	14.40 ± 0.02 ^Bd^	16.54 ± 0.05 ^Ae^	13.47 ± 0.51 ^Be^
	2	23.63 ± 0.09 ^Cd^	27.61 ± 0.81 ^Bb^	18.90 ± 0.45 ^Dc^	29.61 ± 0.03 ^Ab^
	4	45.83 ± 0.16 ^Ac^	41.50 ± 0.18 ^Ba^	35.84 ± 0.57 ^Cb^	31.77 ± 0.40 ^Da^
	6	53.96 ± 0.02 ^Ab^	23.88 ± 0.93 ^Cc^	17.84 ± 0.47 ^Dd^	27.03 ± 1.71 ^Bc^
	8	66.69 ± 0.48 ^Aa^	9.32 ± 0.20 ^De^	42.72 ± 0.25 ^Ba^	21.93 ± 0.25 ^Cd^
Tyramine	0	20.73 ± 1.30 ^Bb^	20.36 ± 0.05 ^Ba^	23.9 ± 0.20 ^Aa^	18.56 ± 0.89 ^Ca^
	2	13.77 ± 0.50 ^Ac^	8.03 ± 0.31 ^Bc^	8.24 ± 0.98 ^Be^	7.75 ± 0.44 ^Bc^
	4	11.95 ± 0.45 ^Bc^	13.11 ± 0.77 ^Ab^	12.78 ± 0.18 ^ABc^	6.31 ± 0.12 ^Cd^
	6	13.38 ± 0.41 ^Bc^	8.11 ± 0.88 ^Cc^	13.68 ± 0.42 ^Bb^	17.72 ± 1.00 ^Aa^
	8	23.56 ± 2.96 ^Aa^	5.94 ± 0.32 ^Cd^	9.97 ± 0.08 ^Bd^	9.80 ± 0.22 ^Bb^
Spermidine	0	3.25 ± 0.05 ^Cc^	3.2 ± 0.02 ^Cb^	3.43 ± 0.01 ^Bb^	3.59 ± 0.04 ^Aa^
	2	3.58 ± 0.01 ^Ab^	3.22 ± 0.01 ^Db^	3.27 ± 0.02 ^Cd^	3.35 ± 0.02 ^Bbc^
	4	3.18 ± 0.02 ^Dc^	3.65 ± 0.03 ^Ba^	4.03 ± 0.02 ^Aa^	3.26 ± 0.03 ^Cc^
	6	3.63 ± 0.06 ^Ab^	3.06 ± 0.01 ^Cc^	3.38 ± 0.21 ^Bc^	3.39 ± 0.09 ^Bb^
	8	4.14 ± 0.07 ^Aa^	3.11 ± 0.12 ^Cc^	3.42 ± 0.01 ^Bb^	3.39 ± 0.02 ^Bb^
Total BA	0	60.95 ± 5.12 ^Be^	58.41 ± 0.05 ^BCd^	70.63 ± 0.10 ^Ae^	54.38 ± 0.79 ^Ce^
	2	111.44 ± 0.38 ^Dd^	134.45 ± 3.87 ^Bc^	127.18 ± 3.59 ^Cd^	153.75 ± 0.38 ^Ad^
	4	236.37 ± 0.55 ^Bc^	253.62 ± 0.33 ^Aa^	194.76 ± 2.81 ^Cc^	189.39 ± 2.35 ^Dc^
	6	311.08 ± 2.12 ^Ab^	259.23 ± 7.55 ^Ba^	238.50 ± 6.55 ^Cb^	231.77 ± 15.79 ^Cb^
	8	409.62 ± 4.99 ^Aa^	236.63 ± 2.29 ^Db^	280.79 ± 1.53 ^Ba^	253.66 ± 4.22 ^Ca^
Nitrite	0	0.92 ± 0.01 ^Ab^	0.83 ± 0.13 ^Ab^	0.79 ± 0.16 ^Ab^	0.83 ± 0.19 ^Ab^
	2	0.80 ± 0.07 ^Ab^	0.78 ± 0.03 ^Ab^	0.76 ± 0.04 ^Ab^	0.70 ± 0.06 ^Abc^
	4	0.52 ± 0.03 ^Ac^	0.73 ± 0.03 ^Ab^	0.61 ± 0.10 ^Ab^	0.74 ± 0.11 ^Abc^
	6	0.95 ± 0.08 ^Ab^	0.77 ± 0.09 ^ABb^	0.59 ± 0.15 ^Bb^	0.56 ± 0.09 ^Bc^
	8	3.57 ± 0.05 ^Aa^	2.59 ± 0.19 ^Ca^	2.85 ± 0.07 ^Ba^	2.32 ± 0.16 ^Da^

Note: Treatment groups: CK: natural fermentation, LB: *L. brevis* inoculated, SC: *S. cerevisiae* inoculated, LS: *L. brevis* and *S. cerevisiae* co-culture. ^a–e^ Values in the same group with different letters were significantly different (*p* < 0.05), while ^A–D^ Values on the same day with different letters were significantly different (*p* < 0.05).

## Data Availability

Data will be made available on request.
